# Successful Bailout Retrieval of an Active‐Fixation Atrial Leadless Pacemaker Following Suspected Tissue Entrapment During Repositioning

**DOI:** 10.1002/joa3.70483

**Published:** 2026-10-11

**Authors:** Toshiya Yoshida, Akira Kasagawa, Daisuke Togashi, Ikutaro Nakajima, Tomoo Harada

**Affiliations:** ^1^ Department of Cardiology St. Marianna University School of Medicine Kawasaki Japan

## Abstract

An 81‐year‐old woman with a medical history of mechanical mitral valve replacement, Maze procedure, and multiple myeloma presented with symptomatic sinus arrest and underwent AVEIR DR implantation. Although the AVEIR VR was successfully implanted, optimal electrical parameters could not be achieved during AVEIR AR placement. During repositioning of the AVEIR AR, myocardial entrapment with helix stretching was encountered. Here, we report that advancing the protective sleeve to fully encapsulate the device potentially enabled safe and successful retrieval.

## Graphical abstract

(A) Unscrewing of AVEIR AR. (B) After unscrewing, the device appeared to be trapped with the stretched helix. (C) Successful retrieval was achieved by fully covering the device. (D) The retrieved device demonstrated the stretched helix.

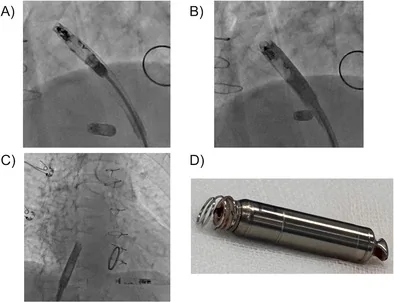

An 81‐year‐old woman was referred for transient visual blackout. Holter monitoring revealed symptomatic sinus arrest with a maximum pause of 8.9 s, consistent with sick sinus syndrome (SSS) type II. She had undergone mechanical mitral valve replacement and Maze procedure and was receiving bisoprolol, warfarin, and lenalidomide for multiple myeloma. Although bisoprolol was discontinued, sinus arrests persisted. No reversible precipitating factors were identified. Given her increased infection risk [1], baseline sinus rhythm, and symptomatic SSS, the dual‐chamber leadless pacemaker (AVEIR DR, Abbott, Abbott Park, IL) was selected.

On hospital day 5, implantation of the AVEIR DR was performed. The procedure was carried out according to the optimized technique [2]. The preprocedural PT‐INR was 1.78, and warfarin (2 mg/day) was continued without heparin bridging, considering both bleeding and thromboembolic risks. AVEIR VR implantation was performed first. Right ventriculography confirmed positioning toward the lower septum (Figure 1A,B), and pre‐mapping showed a COI amplitude > 2 mV with acceptable parameters (Figure 1C). After screw fixation, the pacing threshold, sensed amplitude, and impedance were 2.5 V at 0.4 ms, 5.2 mV, and 510 Ω, respectively. As threshold improvement was anticipated, AVEIR AR implantation was initiated.

**FIGURE 1:**
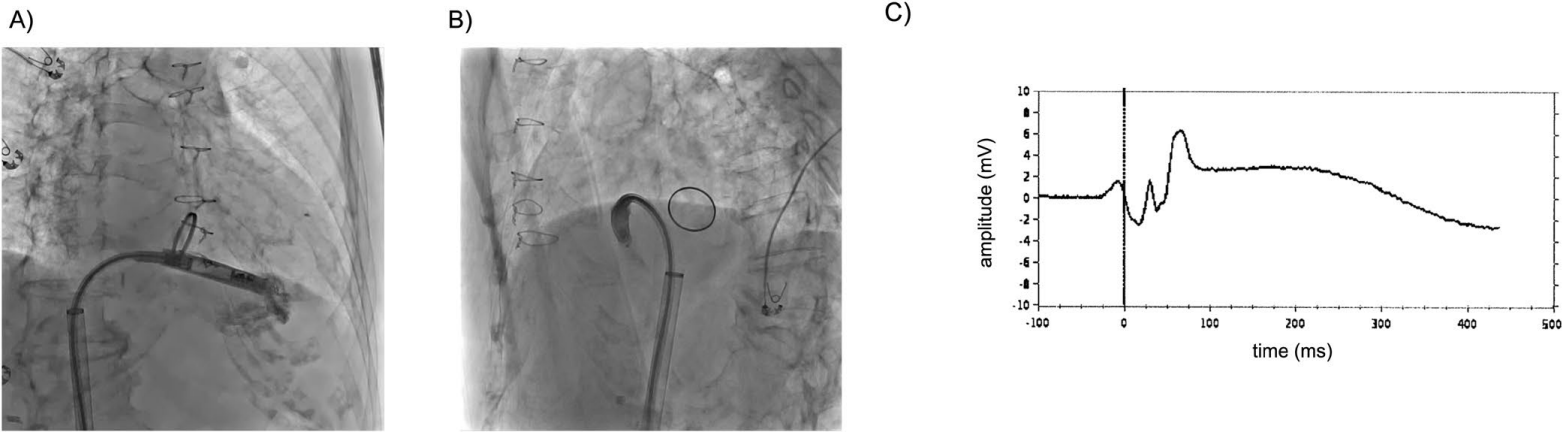
Right ventriculography before implantation of the AVEIR VR and the current of injury amplitude during pre‐mapping. (A) The right anterior oblique 30° view and (B) the left anterior oblique 60° view. (C) Pre‐mapping showed current of injury amplitude exceeded 2 mV.

Right atrial angiography (RAG) confirmed device positioning at the basal lateral right atrial appendage (RAA) (Figure 2A,B and Video S1). The intracardiac electrogram was atypical, and the COI amplitude remained low throughout five mapping attempts (Figure 2C), possibly due to atrial scarring from the prior Maze procedure. The fifth attempt yielded the best parameters: pacing threshold, 1.0 V at 0.4 ms; sensed amplitude, 0.8 mV; and impedance, 240 Ω. Although the latter two were slightly below the recommended targets [2], we proceeded with fixation after team discussion. After a reference cine was recorded (Figure 3A), the stability of the device position was confirmed, and slow clockwise rotation was initiated. Rotation of the chevron marker was observed after the first click. Impedance increased only to 250 Ω, which was considered acceptable. After approximately 1.5 rotations of the device were confirmed fluoroscopically, fixation was completed. The system was then switched to long‐tether mode, and a tension test was performed, confirming satisfactory fixation (Figure 3B and Video S2). However, despite a waiting period, assessment of the COI amplitude remained difficult (Figure 3C). The sensed amplitude was < 0.5 mV, the pacing threshold was 5.5 V at a pulse width of 1.5 ms, and the impedance was 230 Ω. Therefore, repositioning was planned. After the redocking, the protective sleeve was advanced slowly and a reference cine was obtained (Figure 4A), unscrewing was initiated. The control knob was slowly rotated counterclockwise, and the chevron marker completed three full turns. However, neither device rotation nor separation from the endocardium was clearly observed fluoroscopically. Therefore, an additional counterclockwise turn of the knob was applied, followed by a cautious attempt to advance the protective sleeve to fully cover the device. When repositioning was attempted, resistance was encountered, raising suspicion of tissue entrapment, and fluoroscopy demonstrated stretching of the helix (Figure 4B). Gentle clockwise and counterclockwise rotation were attempted, but no change was observed. After team discussion, the device was kept fully covered by the protective sleeve under fluoroscopic guidance. The control knob was then rotated counterclockwise one click at a time with gentle traction. After the second click, resistance disappeared and device mobility was confirmed fluoroscopically, allowing the device to be carefully withdrawn (Video S3). Follow‐up with RAG and TTE demonstrated no pericardial effusion, and the patient remained hemodynamically stable. Reimplantation of the AVEIR AR was considered, but selection of a different location was technically difficult. In addition, the AVEIR VR pacing threshold had improved to 0.5 V at 0.4 ms, providing adequate backup pacing. Given the absence of atrioventricular conduction disturbance on prior Holter monitoring and in‐hospital telemetry, the procedure was concluded without reimplantation of the atrial device. Postprocedural inspection of the retrieved AVEIR AR demonstrated stretching of the helix, with no visible adherent tissue (Figure 4C). Her symptoms resolved with VVI pacing at 60 beats/min, and she remained free of vascular or bleeding complications. After warfarin adjustment, she was discharged home on hospital day 10 with a PT‐INR of 2.01. Close follow‐up was planned for increased ventricular pacing burden or development of atrioventricular block, with alternative strategies to be reconsidered.

**FIGURE 2:**
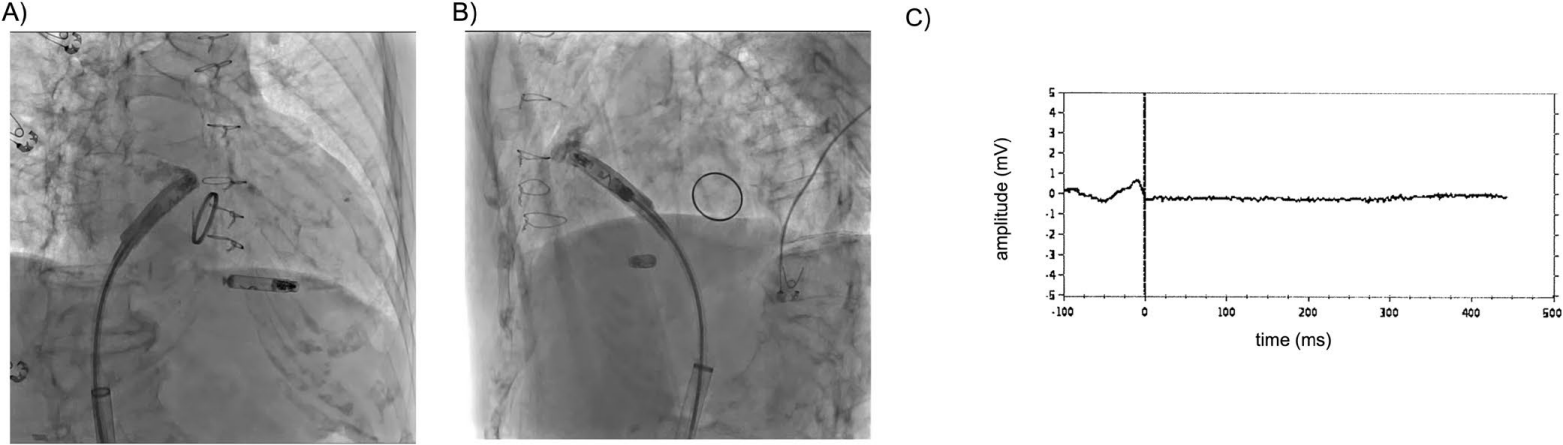
Right atriography before implantation of the AVEIR AR and the current of injury amplitude during pre‐mapping. (A) The right anterior oblique 30° view and (B) the left anterior oblique 60° view. (C) In the pre‐mapping, the waveform of the commanded intracardiac electrogram was atypical and the COI amplitude was low.

**FIGURE 3:**
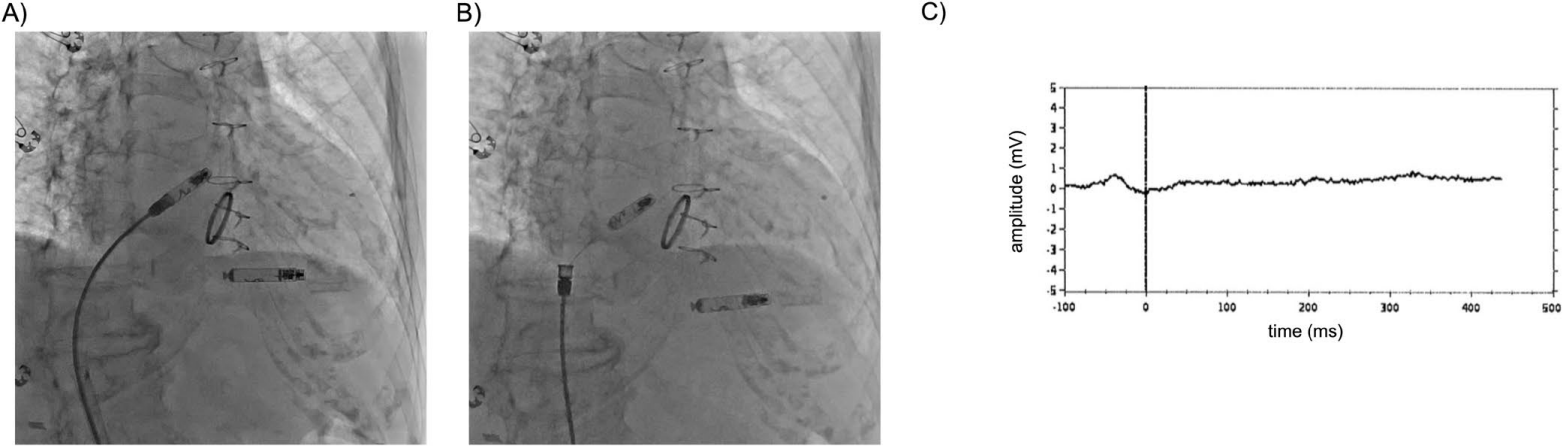
Fixation of AVEIR AR, long‐tether mode and a tension test and the current of injury amplitude after implantation. (A) A reference cine was recorded before fixation. (B) The system was switched to long‐tether mode, and a tension test was performed, confirming satisfactory fixation. (C) The re‐evaluation of the current of injury amplitude remained difficult.

**FIGURE 4:**
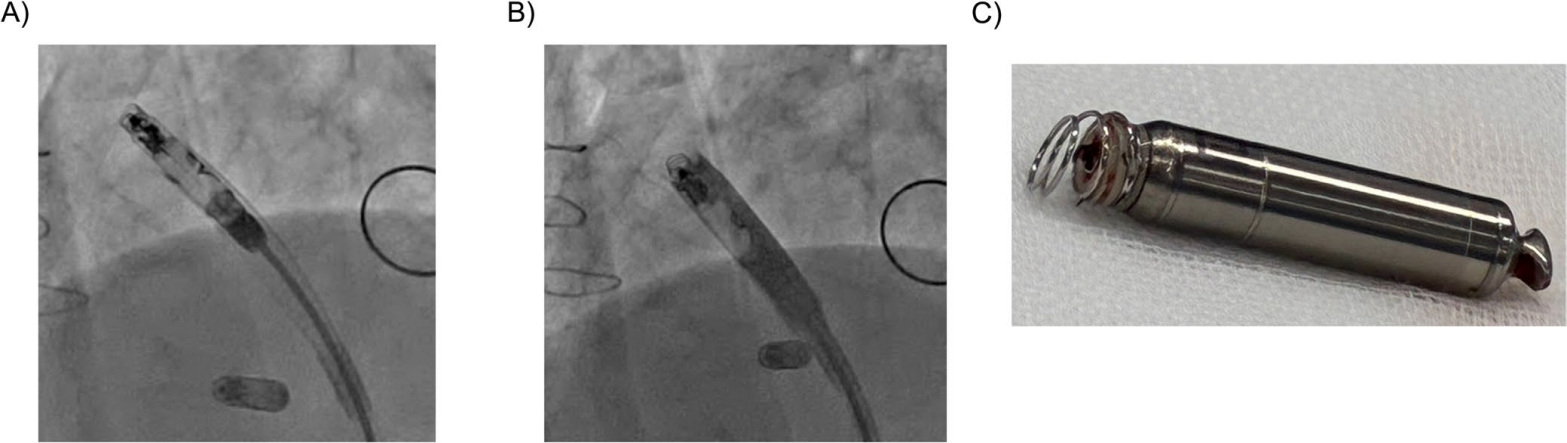
Successful retrieval of the AVEIR AR with suspected entrapment and a stretched helix. (A) After a reference cine was obtained, unscrewing was initiated. (B) After unscrewing, the device appeared to be trapped, and fluoroscopy demonstrated stretching of the helix. (C) Postprocedural inspection of the retrieved AVEIR AR demonstrated stretching of the helix, with no visible adherent tissue.

Previous AVEIR‐related complications include right ventricular perforation, high pacing thresholds, and device dislodgement [1]. Helix‐related complications, including stretched, bent, or broken helixes, have also been reported [3]. However, device entrapment as observed in this case has not been described. Although tissue entrapment was suspected based on retrieval resistance, helix stretching, and tactile release, alternative mechanisms cannot be excluded, including unfavorable device–myocardium alignment and helix deformation due to incomplete torque transmission or excessive manipulation.

A similar phenomenon has been described during deep septal screw‐in lead deployment. Jastrzębski et al. [4] identified four distinct lead behaviors. Although their relevance to the present case remains uncertain, active‐fixation LPs with a similar screw‐in mechanism may also be susceptible to entrapment.

In the present case, a strategy of advancing the protective sleeve to fully cover the device was adopted. The protective sleeve can be readily identified fluoroscopically when it comes into contact with adjacent structures, allowing controlled and atraumatic manipulation. Full distal coverage may also have provided counter‐support analogous to the counter‐traction principle used during transvenous lead extraction [5], thereby facilitating tissue disengagement while minimizing direct traction on the surrounding myocardium. However, deep sleeve advancement may itself cause tissue entrapment or injury. In this case, postoperative pericardial adhesions may have mitigated the consequences of cardiac injury. Thus, the favorable outcome may not be generalizable. Nevertheless, such entrapment may occur during implantation or chronic retrieval, and this strategy may provide a useful bailout option.

## Conclusion

In the present case, AVEIR AR entrapment with a stretched helix was encountered during repositioning. Successful retrieval was achieved by fully covering the device. Although such a complication has not been previously reported and appears to be rare, operators should be aware of its potential occurrence given the screw‐in fixation mechanism.

## Funding

The authors have nothing to report.

## Ethics Statement

This case report complies with the ethics and integrity policies of the Journal of Arrhythmia.

## Consent

Written informed consent was obtained from the patient for publication of this case report and accompanying images.

## Conflicts of Interest

The authors declare no conflicts of interest.

## Supporting information


**Video S1:** Right atriography before fixation of the AVEIR AR. (A) The right anterior oblique 30° view and (B) The left anterior oblique 60° view.

**Video S2:** Long‐tether mode and a tension test after fixation. The system was switched to long‐tether mode, and a tension test was performed, confirming satisfactory fixation.

**Video S3:** Successful retrieval of the AVEIR AR with suspected entrapment and a stretched helix. The device was kept fully covered by the protective sleeve under fluoroscopic guidance. The control knob was then rotated counterclockwise one click at a time with gentle traction. After the second click, resistance disappeared and device mobility was confirmed fluoroscopically, allowing the device to be carefully withdrawn.

## Data Availability

The authors have nothing to report.

## References

[1] Y. Iwasaki , T. Noda , M. Akao , et al., “JCS/JHRS 2024 Guideline Focused Update on Management of Cardiac Arrhythmias,” Circulation Journal 89 (2025): 1012–1073. 10.1253/circj.CJ-24-0073 39956587

[2] P. Neuzil , C. Hubbard , R. Doshi , et al., “Implantation Techniques for a Helix‐Fixation Dual‐Chamber Leadless Pacemaker,” Heart Rhythm 21 (2024): 2552–2562. 10.1016/j.hrthm.2024.09.053 39343120

[3] J. Garg , K. Shah , R. Bhardwaj , et al., “Adverse Events Associated With Aveir VR Leadless Pacemaker: A Food and Drug Administration MAUDE Database Study,” Journal of Cardiovascular Electrophysiology 6 (2023): 1469–1471. 10.1111/jce.15932 37209414

[4] M. Jastrzębski , P. Moskal , M. K. Hołda , et al., “Deep Septal Deployment of a Thin, Lumenless Pacing Lead: A Translational Cadaver Simulation Study,” Europace 1 (2020): 156–161. 10.1093/europace/euz270 31722391

[5] J. Jiratham‐Opas , N. Prasertwitayakij , T. Nantsupawat , and W. Wongcharoen , “Procedural Outcome of Lead Explant and Countertraction‐Assisted Femoral Lead Extraction in Thai Patients With Cardiac Implantable Electronic Device Infection,” Journal of Arrhythmia 5 (2021): 1124–1130. 10.1002/joa3.12574 PMC8485811 34621410

